# A Novel Simulation Model of Shielding Performance Based on the Anisotropic Magnetic Property of Magnetic Shields

**DOI:** 10.3390/ma17235906

**Published:** 2024-12-02

**Authors:** Yuzheng Ma, Minxia Shi, Leran Zhang, Teng Li, Xuechen Ling, Shuai Yuan, Hanxing Wang, Yi Gao

**Affiliations:** 1 Key Laboratory of Ultra-Weak Magnetic Field Measurement Technology, Ministry of Education, School of Instrumentation Science and Optoelectronics Engineering, Beihang University, Beijing 100191, China; 21myz@buaa.edu.cn (Y.M.); 2423660154@buaa.edu.cn (T.L.); zy2243140@buaa.edu.cn (X.L.); ys@buaa.edu.cn (S.Y.); whx0420@buaa.edu.cn (H.W.); gaoyi0509@163.com (Y.G.); 2 Zhejiang Provincial Key Laboratory of Ultra-Weak Magnetic-Field Space and Applied Technology, Hangzhou Innovation Institute, Beihang University, Hangzhou 310051, China; 3 Hefei National Laboratory, Hefei 230088, China

**Keywords:** 2D-SST measuring device, anisotropic magnetic properties, magnetic field simulation, shielding factor (SF), performance calculation model

## Abstract

To achieve a near-zero magnetic field environment, the use of permalloy sheets with high-performance magnetic properties is essential. However, mainstream welding processes for magnetically shielded rooms (MSRs), such as argon arc welding and laser welding, can degrade the magnetic properties of the material. Additionally, neglecting the anisotropy of permalloy sheets can introduce unpredictable errors in the evaluation of MSR performance. To address this issue, this paper proposes a modified model for calculating the shielding factor (SF) of MSRs that incorporates the anisotropic magnetic characteristics of permalloy sheets. These characteristics were measured using a two-dimensional single sheet tester (2D-SST). A high-precision measurement system was developed, comprising a 2D-SST (to generate two-dimensional magnetic fields and sense the induced *B* and *H* signals) and a control system (to apply in-phase 2D excitation signals and amplify, filter, and record the *B* and *H* data). Hysteresis loops were tested at low frequencies (0.1–9 Hz) and under different magnetization states (0.1–0.6 T) in two orientations—parallel and perpendicular to the annealing magnetic field—to verify anisotropy under varying conditions. Initial permeability, near-saturation magnetization, and basic magnetization curves (BM curves) were measured across different directions to provide parameters for simulations and theoretical calculations. Based on these measurements and finite element simulations, a mathematical model was developed to adjust the empirical coefficient *λ* used in theoretical SF calculations. The results revealed that the ratio of empirical coefficients in different directions is inversely proportional to the ratio of magnetic permeability in the corresponding directions. A verification group was established to compare the original model and the modified model. The mean squared error (MSE) between the original model and the finite element simulation was 49.97, while the MSE between the improved model and the finite element simulation was reduced to 0.13. This indicates a substantial improvement in the computational accuracy of the modified model.

## 1. Introduction

A nearly zero magnetic field environment [1] with high uniformity and low temporal drift is a precondition for different research [2,3], such as atomic gyroscopes [4,5] and magnetometers [6,7], in which the extremely low magnetic field is the precondition for an atomic sensor [8] to enter the Spin-Exchange Relaxation Free Regime (Serf) state [9] and the guarantee of high precision sensitivity [10,11]. In medical and biomedical science [12], in the research on weak biomagnetic signal recording, such as magnetoencephalography and magnetocardiograms [13,14], the target signal is extremely weak and easily disturbed by surrounding magnetic interference. The magnetically shielded rooms (MSRs), which are usually composed of high-conductivity materials to realize an external magnetic field, rely on the eddying and flux shunt effects, respectively, and can provide the magnetic field environment needed by the above research [15,16]. The shielding factor (SF) represents the shielding performance of MSRs, which is directly determined by the permeability of permalloy sheet [17].

Physikalisch–Technische Bundesanstalt introduces the basic structure, assembly process, and shielding performance of cube-MSR [18,19]. Sun et al. proposed a numerical model to calculate the SF and optimize the structure for MSRs with the permeability tested and equivalent corrected [20,21]. Yang et al. applied a weak excitation on MSRs to change the permeability for the improvement of the SF [22]. Shi et al. measured the magnetic property of a permalloy sheet in low-frequency and degaussing excitation, which enhanced the accuracy of calculations and simulations [23]. In the previous research on the performance of MSRs, permeability is usually used as a number, which means permeability is regarded as the same in all directions. However, the structure of permalloy is cubic crystal, in which the Ni and Fe will merge into pairs of homogeneous atoms or chains of homogeneous atoms in a certain direction when permalloy is magnetic and annealing below the Curie temperature [24,25], which leads to the magnetic property in each direction being obviously different. The permalloy is easily magnetized in the direction following magnetic annealing, and it is hard to magnetize in the direction perpendicular to the magnetic annealing, and the degree of magnetization difficulty is medium when the direction is the median of the above two directions. Therefore, the permeability used in the simulation and calculation of MSRs should be anisotropic rather than isotropic.

The International Electro Technical Commission (IEC) has proposed a single sheet tester (SST) which is revised repeatedly and regarded as the standard for magnetic property measurement. The B-H sensor for a permalloy sheet usually consists of a B probe and H coil [26,27], which can supply splendid measurement accuracy while having no influence on the physical structure of the test specimen. At present, the measurements of the magnetic characteristics of permalloy sheets are various, such as with specific excitation [22], in a state of anhysteric [28], under demagnetizing excitation [29], and in different temperature fields [30]. However, the above magnetic property was measured on a test ring or in a single direction, which ignores the anisotropy of permalloy. The two-dimensional single sheet tester (2D-SST) usually measures the magnetic properties and hysteresis loss of non-oriented electrical steel under different temperatures, stress loading, and excitation field directions, which are used for the design and performance calculation of magnetic core [31,32]. The measurement of magnetic anisotropy of permalloy sheets for MSR design is rare, therefore, the calculation empirical formula of the SF for the cube-MSR is inaccurate and uses a single permeability.

In this paper, a two-dimensional single sheet tester (2D-SST) is established to measure the anisotropic permeability of a permalloy sheet for improving the simulation accuracy of MSRs, based on the measurement data and finite element simulation to propose a modified model to optimize the empirical formula for the SF of the cube-MSR. First, the permeability in directions parallel and perpendicular to the magnetic annealing of the permalloy sheet are tested in different frequencies (0.1~10 hz), excitation, and welding process (argon arc welding and laser welding) to analyze the otherness of anisotropy in these cases. Furthermore, the finite element simulations of magnetic field distribution in isotropic permeability and anisotropic permeability (in argon arc welding and laser welding) are compared and analyzed. Finally, based on the simulation result of SF in each direction, the mathematical correlation between the empirical coefficient *λ* and anisotropic permeability is found, and the modified model for optimizing the accuracy of the theoretical calculation of the shielding factor in the anisotropic case is established.

The remainder of this paper is organized as follows. In Section 2, the principle, physical structure, and instrumentation of the measurement system based on the 2D-SST are introduced. Section 3 presents the measurement data of anisotropic permeability in various directions and measures further magnetic properties for subsequent simulations. Section 4 establishes a modified model and quantifies the mathematical relationships for the empirical coefficient *λ* and anisotropic permeability based on the simulation result. Section 5 concludes this paper.

## 2. The Design and Application of 2D-SST

**Figure 1:**
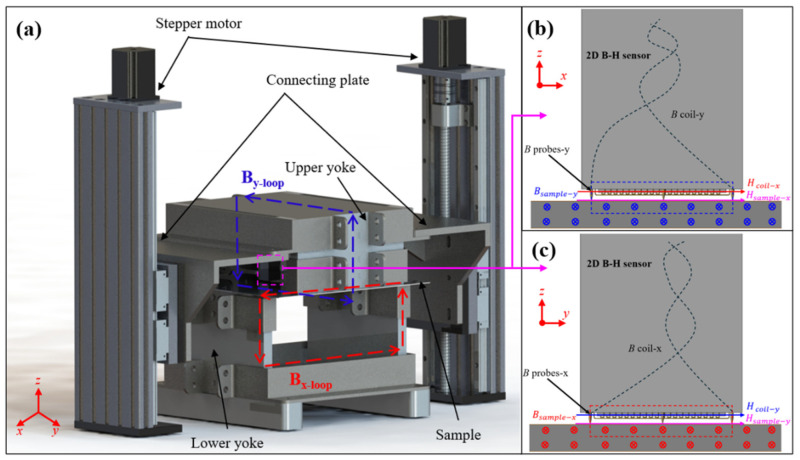
The desired field and the feedback measurement system: (**a**) desired field; (**b**) schematic of the experimental measurement system; (**c**) the physical single sheet tester (SST).

The measurement system consists of a 2D-SST (self-innovation), which includes a two-dimensional B-H sensor, along with signal generation, filtering, and recording equipment. The basic structure of the 2D-SST comprises two U-shaped magnetic yokes with wound excitation coils, the specimen of the permalloy sheet, and a two-dimensional B-H sensor, which is shown in Figure 1a. The U-shaped magnetic yokes are assembled from multiple non-grain-oriented silicon steel sheets to minimize eddy current effects associated with highly conductive metallic materials. The specimen is clamped by the lower yoke and upper yoke, which are supported and controlled by two stepper motors through connecting plates, and the measurement sensor is pressed flat against the center surface of the sample. The spatially perpendicular yokes and the specimen constitute two mutually perpendicular closed magnetic circuits, ensuring the uniformity of the magnetic field in the central region of the permalloy sheet based on achieving anisotropic measurements.

The two-dimensional B-H sensor consists of a two-dimensional *B* probe-*B* coil and a two-dimensional *H* coil. As illustrated in Figure 1b,c, the closed loops formed by the *B* probes, *B* coils, and the test specimen induce voltages when the specimen undergoes magnetization. According to Faraday’s law of electromagnetic induction, the magnetic flux density can be calculated using the following equation.

(1) $$B_{sample}=-\frac{1}{N_{B}S_{B}}\int V_{B}dt$$

where *V_B_* represents the induced voltage between probes in each direction, *S_B_* is the cross-sectional area of the measured area, and *N_B_* is equivalent to 0.5 for the probe method, since only half of the flux passes through the sensing coil when the magnetic field distribution is relatively uniform inside the permalloy sheet.

The *H* coil, which is composed of multiple turns of enameled copper wire wound around a non-magnetic thin sheet framework in two mutually perpendicular directions, is positioned close to the surface of the specimen, as shown in Figure 1b,c. Based on the boundary condition of continuity for the tangential components in different media and Faraday’s law of electromagnetic induction, the *H_coil_* in the *H* coil can be considered equivalent to the *H_sample_* at the surface of the test specimen, and can be calculated using the following equation:

(2) $$H_{sample}=-\frac{1}{\mu_{0}N_{H}S_{H}}\int V_{H}dt$$

where *V_H_* indicates the induced voltage of the *H* coil in each direction, and *N_H_* is 1000, which represents the turn number of the *H* coil. *S**_H_*** is the cross section of *H* coil, and *μ*_0_ is air permeability.

**Figure 2:**
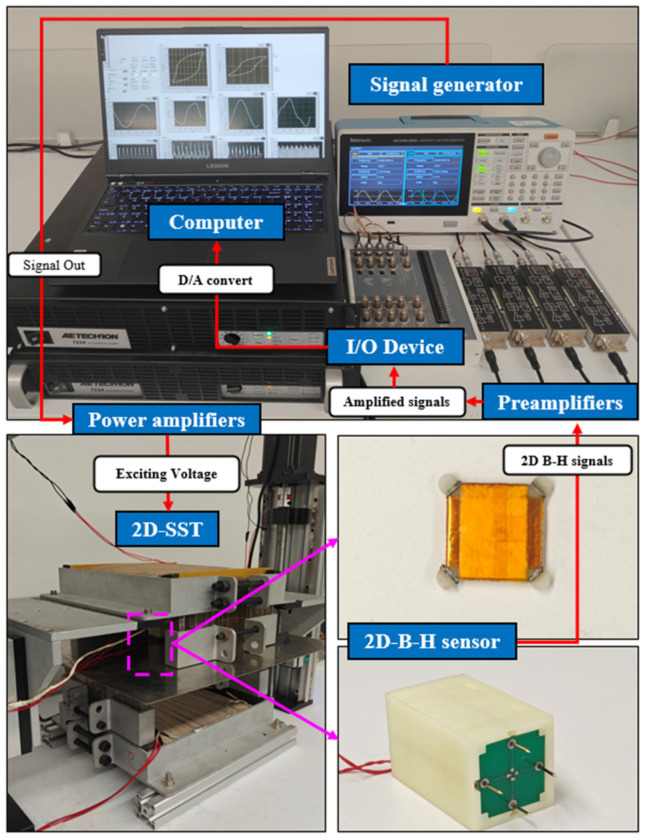
The prototype of the 2D-SST measurement system.

The control system of this measurement apparatus is illustrated in Figure 2. The high-precision signal generator, Tektronix AFG31102 (Tektronix, Beaverton, OR, USA), simultaneously output two excitation signals with a controllable phase difference. The exciting signals are amplified by two AE Techron 7234 (AE Techron, Elkhart, IN, USA) power amplifiers with a gain of 20 and then applied in the exciting coils wound on U-yokes. The 2D B-H signals are filtered and amplified by FEMTO DELPHA preamplifiers (FEMTO, Berlin, Germany) to enhance the signal-to-noise ratio and transmitted to the host computer via a 16-bit NI USB-6366 I/O multifunction device (Emerson, Austin, TX, USA). Within the host computer, the signals are filtered by FFT to further extract the effective signal, followed by mathematical operations to calculate the B-H values and plot the corresponding hysteresis loop.

## 3. The Measurement and Analysis of Anisotropic Magnetic Properties

### 3.1. The Measured Anisotropic Magnetic Properties

To preliminarily measure the basic magnetic anisotropy characteristics of permalloy with two welding processes, this study measured the hysteresis loops under different magnetization states and frequencies, both parallel and perpendicular to the magnetic annealing direction. The magnetization status ranged from the initial magnetization to the saturation stage, with a magnetic induction intensity from 0.1 T to 0.7 T in increments of 0.1 T. The frequencies were focused on the low-frequency range and are the major frequency band of permalloy to shield; they were selected at 9 Hz, 4 Hz, 1 Hz, 0.5 Hz, and 0.1 Hz for avoiding interference from the power line frequencies.

**Figure 3:**
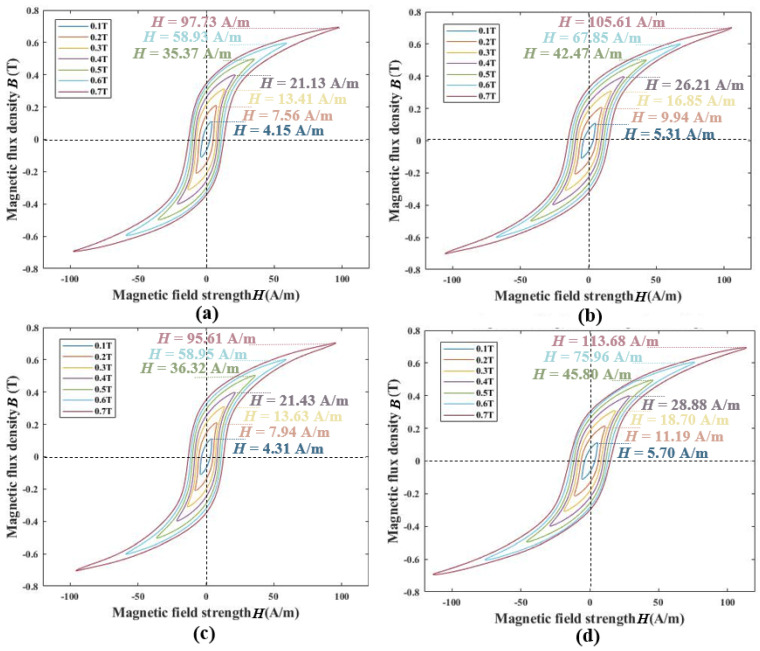
The anisotropic hysteresis loop of B = 0.1~0.7. (**a**) Argon arc welding—parallel to magnetic annealing direction, (**b**) argon arc welding—perpendicular to magnetic annealing direction, (**c**) laser welding—parallel to magnetic annealing direction, (**d**) laser welding—perpendicular to magnetic annealing direction.

As shown in Figure 3a,b, the magnetic field strength *H* of the argon arc welding evolves from 4.15 A/m to 97.73 A/m, and from 5.31 A/m to 105.61 A/m, in directions parallel and perpendicular to magnetic annealing, respectively, while the magnetic flux density *B* shifts from 0.1 T to 0.7 T. For laser welding, the *H* evolves from 4.31 A/m to 95.61 A/m, and from 5.70 A/m to 113.68 A/m, in directions parallel and perpendicular to magnetic annealing, respectively. The permalloy is more readily magnetized in the direction parallel to the magnetic annealing direction compared to the perpendicular direction at each magnetic flux density. Therefore, this demonstrates that permalloys welded by argon arc welding and laser welding processes both exhibit significant anisotropy. The most significant impact of anisotropy on the hysteresis loops under different magnetization states is the variation in the magnetic field strength *H* required to reach the same magnetization intensity *B*. Due to the limited domain reversal speed of permalloy, when the magnetic domains are unable to respond quickly to changes in the applied magnetic field, a lag effect occurs. As the frequency increases, the hysteresis phenomenon intensifies, resulting in a broader hysteresis loop. The anisotropy of the material further reduces the reversal speed of the domains along the difficult-to-magnetize axis.

**Figure 4:**
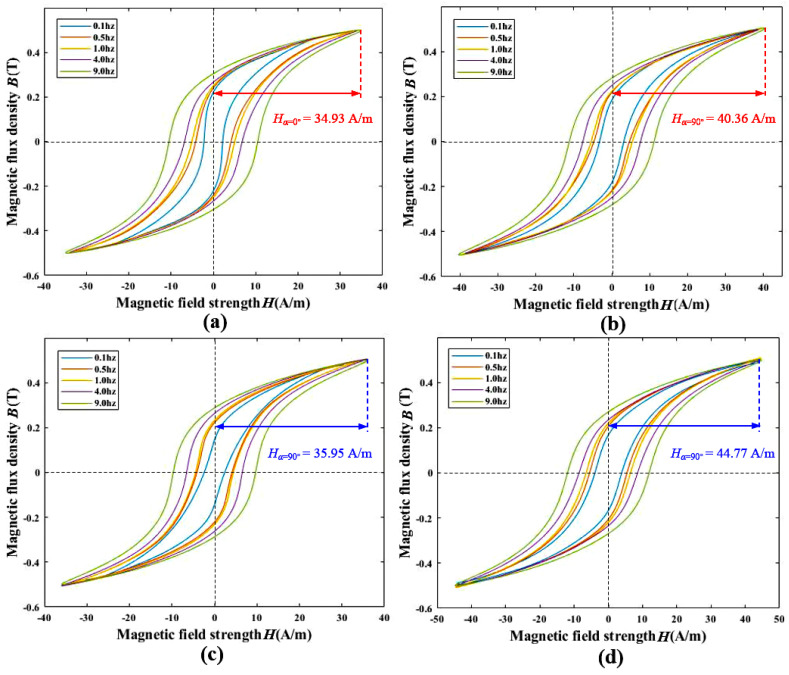
The anisotropic hysteresis loops at low-frequency band. (**a**) Argon arc welding—parallel to magnetic annealing direction, (**b**) argon arc welding—perpendicular to magnetic annealing direction, (**c**) laser welding—parallel to magnetic annealing direction, (**d**) laser welding—perpendicular to magnetic annealing direction.

The result of the measurement for the permalloy sheet at various frequencies at B = 0.5 T is shown in Figure 4. The area of the hysteresis loop gradually increased with the frequency, which indicates the hysteresis losses rise with increasing frequency. The magnetic field strength H in 0.1~9 hz is around 34.93 A/m in the direction parallel to magnetic annealing, while the perpendicular is around 40.36 A/m. For laser welding, the parallel and the perpendicular are 35.95 A/m and 44.77 A/m, respectively. The permalloy in argon arc welding and laser welding exhibit significant anisotropy in the low-frequency range, and are the primary frequency band for the quasi-static geomagnetic field, the electromagnetic interference and mechanical vibrations in the MSR operational environment, and the target signal frequency range in medical and biomedical science.

Based on the above measurement experiments, whether for argon arc welding permalloy or laser welding permalloy, the magnetic properties along the direction parallel to the magnetic annealing orientation are superior to those perpendicular to it, across different magnetization levels and frequencies. At *B* = 0.1 T, argon arc welding permalloy is more easily magnetized compared to the laser welding permalloy. However, as the degree of magnetization increases, at *B* = 0.7 T, the laser welding permalloy becomes easier to magnetize in the direction parallel to the magnetic annealing orientation, while the argon arc welding permalloy is easier to magnetize in the perpendicular direction. In the low-frequency range, both argon arc welding and laser welding permalloys exhibit anisotropy. Along the parallel direction, the *H* required to reach 0.5 T is significantly higher for the laser welding permalloy than for the argon arc welding permalloy. Therefore, the overall anisotropic magnetic performance of argon arc welding permalloy is superior to that of the laser welding permalloy.

### 3.2. Measurement and Comparison of Permalloy Sheet

The measurement of the anisotropic magnetic properties in directions which are parallel and perpendicular to the magnetic annealing direction has initially verified that the permalloy sheets under argon arc welding and laser welding exhibit significant magnetic anisotropy in different magnetization states. For further exploring the magnetic anisotropy of permalloy, the measurement system outputs two excitation signals simultaneously, which apply mutually orthogonal exciting magnetic fields to the samples. By adjusting the amplitude ratio of the two signals, the direction of the total excitement magnetic field can be controlled to measure magnetic anisotropy at more angles. In the subsequent measurements of magnetic anisotropy, the direction which is parallel to the magnetic annealing is regarded as 0°, and the perpendicular is regarded as ±90°.

To explore the variation trend of the magnetic property from the parallel to the vertical in permalloy sheets which were processed by argon arc welding and laser welding, the magnetized states were measured at angles from −90° to 90° with a step size of 15° in excitation magnetic fields of 20 A/m and 40 A/m at 9 Hz.

**Figure 5:**
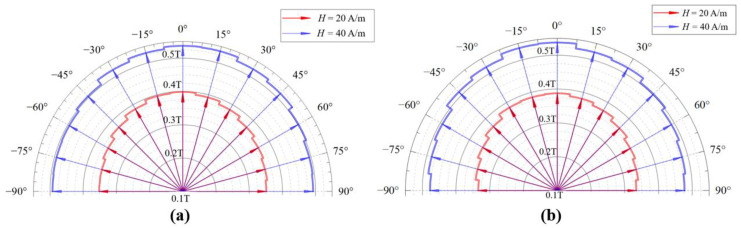
Magnetized states in various directions under excitation magnetic fields of 20 A/m and 40 A/m at 9 Hz. (**a**) Argon arc welding. (**b**) Laser welding.

As shown in Figure 5, the magnetic induction density(B) expands symmetrically and decreases monotonically from the center of the magnetic annealing direction to both sides, which are perpendicular under ammagnetic field intensity of 20 A/m and 40 A/m. This confirms that magnetic anisotropy varies progressively rather than abruptly on both sides of the annealing direction. From Figure 5, the permalloy plate processed by the argon arc welding is more easily magnetized compared to that processed by laser welding.

To investigate the anisotropic variation of permalloy plates throughout the entire magnetization process, the basic magnetization curves of permalloy plates processed by argon arc welding and laser welding were measured from 0° to 90° with a step size of 30°.

**Figure 6:**
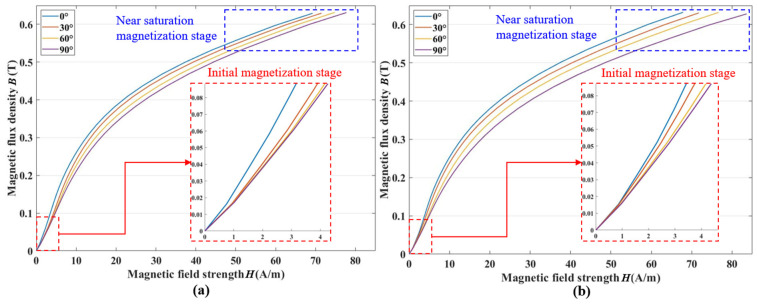
Fundamental magnetization curve. (**a**) Argon arc welding. (**b**) Laser welding.

The test results are shown in Figure 6, and the permalloy plates both exhibit significant anisotropy throughout the entire magnetization process. When *B* shifts from 0.01 T to 0.09 T, the *H* of laser welding evolves from 0.558 A/m to 3.503 A/m in the direction of 0° and from 0.581 A/m to 4.136 A/m in the direction of 90°; for the argon arc, the *H* evolves from 0.464 A/m to 3.129 A/m in the direction of 0° and from 0.590 A/m to 4.227 A/m in the direction of 90°. When *B* shifts from 0.56 T to 0.63 T, the *H* of the laser welding evolves from 51.496 A/m to 67.837 A/m in the direction of 0°, and from 65.204 A/m to 83.586 A/m in the direction of 90°. For argon arc welding, the *H* evolves from 51.790 A/m to 69.259 A/m in the direction of 0°, and from 69.259 A/m to 77.728 A/m in the direction of 90°. The anisotropy of the permalloy plate welded by argon arc welding is more pronounced compared to that of the laser welding plate in the initial stages, and as the degree of magnetization increases to near the saturation magnetization stage, the otherness of anisotropy in the laser welding permalloy plate surpasses that of the argon arc welding plate.

The permalloy is used in MSRs against static and quasi-static low-frequency magnetic fields with a non-hysteretic state in ideal conditions, while the non-hysteretic state is extremely difficult to achieve and maintain in practical applications. The initial permeability generally refers to the magnetization ability exhibited by the permalloy under very weak external magnetic fields. In typical scenarios of MSR use, the external magnetic fields primarily consist of the geomagnetic field, electromagnetic interference from other electrical equipment, and magnetic disturbances caused by mechanical vibrations. These magnetic fields are similar to the external magnetic fields associated with initial permeability. Therefore, the initial permeability (i.e., permeability at a low magnetization state) is commonly used as the parameter to calculate the performance of MSRs and the initial permeability were measured at B = 0.08 T for subsequent simulation in this study.

**Figure 7:**
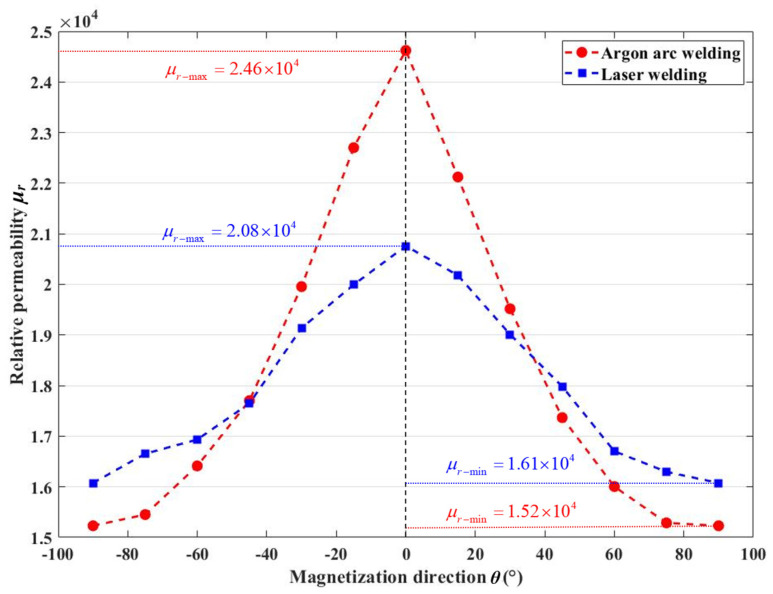
Anisotropic initial permeability.

The measurement results are shown in Figure 7. For argon arc welding permalloy, the *μ_r-max_* = 2.46 × 10^4^ and *μ_r-min_* = 1.52 × 10^4^, and for laser welding permalloy, the *μ_r-max_* = 2.08 × 10^4^ and *μ_r-min_* = 1.61 × 10^4^. As the conclusion in Figure 6, the anisotropy of argon arc welding permalloy was more significant in the initial stages, and the argon arc welding permalloy qualified a more excellent permeability in the direction of 0°.

## 4. Analysis and Optimization of the Calculation for MSR Performance

### 4.1. Simulation of Remanence and SF for MSR

**Figure 8:**
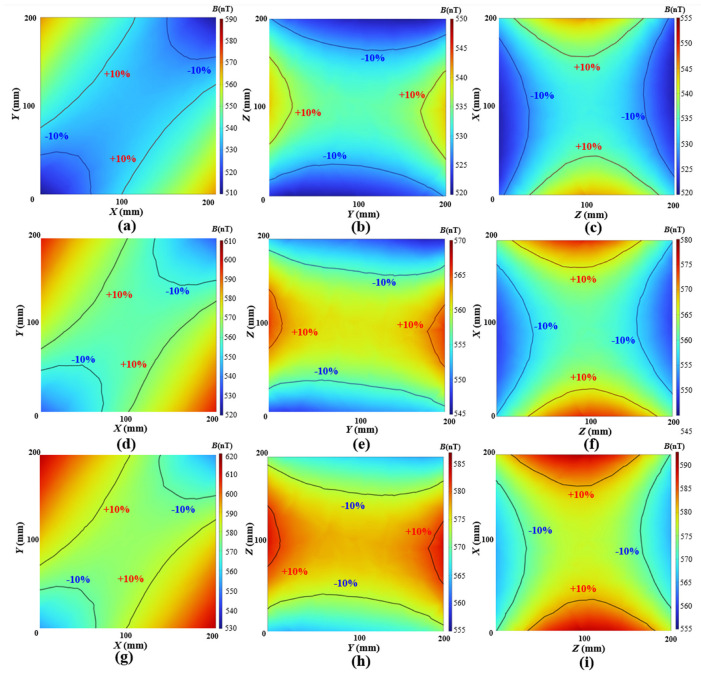
Simulation results of magnetic field distribution. (**a**–**c**) Isotropic permeability μ = 20,000 -xoy plane, yoz plane, and zox plane. (**d**–**f**) Argon arc welding—xoy plane, yoz plane, and zox plane. (**g**–**i**) Laser welding—xoy plane, yoz plane, and zox plane.

To simulate the shielding performance of the MSR made from permalloy with anisotropic magnetic permeability under a low-frequency magnetic field, the test data from the sample in argon arc welding and laser welding were introduced into a finite element model for simulation using COMSOL software 6.1. An infinite domain was established with an applied background geomagnetic field of *B_x_* = 35,117 nT, *B_y_* = 31,202 nT, *B_z_* = 3280 nT, and the MSR was set to 420 × 420 × 420 mm with a thickness of 2 mm. The anisotropic magnetic conductivity along the *x*–axis set the permeability parallel to the magnetic annealing, while the *y*–axis was set perpendicular to it. For the permalloy welded by argon arc welding, the permeabilities were set to *μ_r-x_* = 24,623 and *μ_r-y_* = 15,230, while for the laser welding permalloy the permeabilities were set to *μ_r-x_* = 20,757 and *μ_r-y_* = 16,222, and the *μ_r-z_* were uniformly set to 20,000 in both cases. To compare the simulation results of the shielding performance for the MSR under isotropic and anisotropic magnetic permeability, three control groups with isotropic magnetic permeability were added, in which the permeability values were set to 25,000, 20,000, and 15,000, respectively. The magnetic field in the xoy, yoz, and zox planes of a cubic space, which were located at the center of the shielding device with a size of 200 mm × 200 mm × 200 mm, was recorded and analyzed. The distribution and fluctuation ratio of the magnetic field were relatively consistent in isotropic permeability. Therefore, the simulation results are illustrated in Figure 8 with *μ* = 20,000 of isotropic.

**Figure 9:**
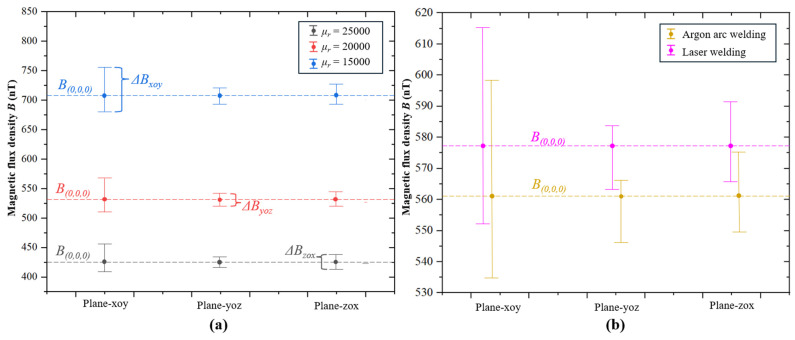
Fluctuation range of magnetic field and origin field strength. (**a**) Isotropic permeability. (**b**) Anisotropic permeability.

To compare the differences of the magnetic field with isotropic and anisotropic magnetic permeability, the magnetic field fluctuation rate on each plane and the shielding factor (SF) at the midpoint were selected as reference metrics. The fluctuation for a plane is defined as the difference between the maximum and minimum magnetic induction intensity, which is expressed as Δ*B*_plane_ = *B*_plane-max_ − *B*_plane-min_ and shown in Figure 9, and the magnetic field fluctuation rate can be calculated by *δ*_plane_ = Δ*B*_plane_/*B* (0, 0, 0), where *B* (0,0,0) is the magnetic induction intensity of the origin. The shielding factor SFi is calculated by SF_i_ = *B*_BGF-i_/*B*_i_ (0, 0, 0), where the BBGF-i is the background geomagnetic field in the direction of x, y, or z, and the *B*_i_ (0, 0, 0) is the magnetic induction intensity of the origin in the corresponding direction.

The evaluation metric results are shown in Table 1. In simulations with isotropic magnetic permeability, the magnetic field fluctuation rate *δ* on each plane remains nearly identical when the magnetic permeability is varied, while other simulation conditions are held constant. In the case of anisotropic permeability, the *δ_xoy_* and *δ_yo__z_* vary observably while the permeability is varied. The shielding factors for isotropic permeability remain essentially uniform across all directions, whereas anisotropic shielding factors vary from 22.8% to 45.7% in argon arc welding, and vary from 17.8% to 21.9% in laser welding.

### 4.2. Analysis and Optimization of Theoretical Calculation for SF

The geomagnetic field primarily consists of static magnetic fields or low-frequency magnetic fields, and the most biological magnetic field signals are below 100 Hz, therefore the magnetic field can be regarded as a static or quasi-static magnetic field which can neglect the influence of eddy currents within the shielding material, then the shielding problem will be transformed into a boundary value problem for static magnetic fields in different media. The SFs for spherical and infinitely long cylindrical magnetic shielding are solved by a continuity equation for the magnetic scalar potential of the shielding layer. According to Maxwell’s equations, in regions without current, the *H* and *B* can be expressed as:

(3) ∇×H

(4) ∇·B=0

Introducing the magnetic scalar potential *W*, the following condition can be obtained:

(5) H=−∇W

(6) ∇·B=∇·(μ∇W)=0

where the *μ* is magnetic conductivity, and the above expression can be written as:

(7) $$\nabla^{2}W=0$$

The above equation represents the Laplace equation for the magnetic scalar potential, the general solution of which is a harmonic function. In cylindrical and spherical coordinates, it can be expressed as follows:

(8) $$W(r,\theta )=\sum_{n=1}^{\infty }\left[r^{n}(A_{n}\sin n\theta +B_{n}\cos n\theta )+r^{-n}(C_{n}\sin n\theta +D_{n}\cos n\theta )\right]$$

(9) $$W(R,\theta )=\sum_{n=1}^{\infty }\left(a_{n}R^{n}+\frac{b_{n}}{R^{n+1}}\right)P_{n}(\cos\theta )$$

where the *P_n_*(cos*θ*) is the Legendre functions, *P*_0_ = 1, *P*_1_(cos*θ*) = cos*θ*.

The outer and inner diameters of the spherical shielding device are set as *R*_1_ and *R*_2_, respectively. The magnetic scalar potential within each medium satisfies the Laplace equation, and the magnetic scalar potential in each section, can be calculated using the following formulas:

(10) $$\begin{array}{l} W_{0}=(-H_{0}\cdot R+\frac{I_{0}}{R^{2}})\cos\theta (R>R_{1})\\ W_{1}=(-H_{1}\cdot R+\frac{I_{1}}{R^{2}})\cos\theta (R_{1}>R>R_{2})\\ W_{2}=-H_{2}\cdot R\cos\theta (R_{2}>R) \end{array}$$

Based on the boundary conditions that the normal component of the magnetic flux density *B* and the tangential component of the magnetic field intensity *H* are continuous at the interfaces between different media, the following equations can be obtained:

(11) $$\begin{array}{l} W_{0}=W_{1},\frac{\partial W_{0}}{\partial R}=\mu \frac{\partial W_{1}}{\partial R}\\ W_{1}=W_{2},\frac{\partial W_{1}}{\partial R}=\mu \frac{\partial W_{2}}{\partial R} \end{array}$$

To solve the above equations simultaneously, the following equations can be obtained:

(12) $$\left(\begin{array}{c} -H_{0}\\ I_{0} \end{array}\right)=A_{1}A_{2}\left(\begin{array}{c} -H_{2}\\ 0 \end{array}\right)$$

where the *A*_1_ and *A*_2_ are:

(13) $$\begin{array}{l} A_{1}=\left(\begin{array}{cc} \frac{\mu +2}{3} & \frac{2(1-\mu )}{3}\cdot \frac{1}{R_{1}^{3}}\\ \frac{1-\mu }{3}\cdot \frac{1}{R_{1}^{3}} & \frac{2\mu +1}{3} \end{array}\right)\\ A_{2}=\left(\begin{array}{cc} \frac{\mu^{-1}+2}{3} & \frac{2(1-\mu^{-1})}{3}\cdot \frac{1}{R_{1}^{3}}\\ \frac{1-\mu^{-1}}{3}\cdot R_{1}^{3} & \frac{2\mu^{-1}+1}{3} \end{array}\right) \end{array}$$

The shielding factor (SF) can be obtained by calculating *A*_1_*A*_2_.

(14) $$S_{sphere}=\frac{H_{outside}}{H_{inside}}=1+\frac{2}{9}\cdot \frac{{\left(\mu -1\right)}^{2}}{\mu }\cdot (1-\frac{R_{2}^{3}}{R_{1}^{3}})$$

The above equation can be simplified while the magnetic permeability *μ_r_* is significantly greater than air and the thickness *d* of the shielding layer is much less than the outer radius *r*.

(15) $$S=1+\frac{2}{3}\frac{\mu_{r}d}{r}$$

Since the boundary conditions are difficult to express, the shielding factor is not feasible to analytically calculate for a cubic magnetic shielding device. The shielding factor of cubic MSR is usually calculated by an empirical formula.

(16) $$S_{\mathrm{square}}≅1+\lambda \cdot \mu \cdot \frac{d}{R_{1}}\lambda \in \left[0.8,0.96\right]$$

**Figure 10:**
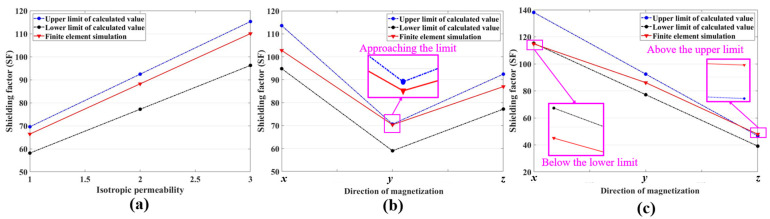
Comparison of theoretical calculated values with finite element simulation for SF. (**a**) Isotropic permeability. (**b**) Anisotropic permeability—argon arc welding. (**c**) Anisotropic permeability—lasers welding.

To investigate the applicability of the theory calculation Formula (16) in isotropic and anisotropic magnetic permeability, the theoretical upper and lower bounds of SF were calculated for isotropic permeability, argon arc welded permalloy, and a simulation group with more significant anisotropic magnetic permeability, which is *μ_x_* = 30,000, *μ_y_* = 20,000, and *μ_z_* = 10,000. And the theoretical results were validated through finite element simulations which are shown in Figure 10.

For isotropic permeability, the simulation results fall within the calculated range, which is shown in Figure 10a, and the empirical coefficient *λ* obtained from the simulation values is consistently 0.9156, whether the permeability is 15,000, 20,000, or 25,000. The fixed *λ* demonstrates the isotropic permeability has no effect on *λ*, while the other conditions are constant. In Figure 10b,c, the SFs along the three coordinate axes in MSRs with anisotropic permeability either approximate or exceed the theoretical upper and lower bounds, which indicates the permeabilities have a significant impact on the theoretical calculation of SF in the case of anisotropy. The empirical coefficients *λ_x_*, *λ_y_*, and *λ_z_* of argon arc welding obtained from the simulation values are 0.8678, 0.9564, and 0.9029 for the simulation group, and the *λ_x_*, *λ_y_*, and *λ_z_* are 0.7968, 0.8937, and 0.9867, respectively. Permeability has an impact on *λ* during the calculation process involving magnetic anisotropy, which leads to considerable errors in the theoretical calculation of the shielding model when a fixed *λ* is used. Therefore, this study improves the empirical model by modifying the one-dimensional calculation formula of SF to a three-dimensional format and incorporating a correction function to optimize *λ*.

For constructing the model, a database is established and shown in Table 2, which includes the permeability of common grades of permalloy alloys, and the permeability under various magnetization states. The *λ* in Table 2. are calculated by finite element simulation in corresponding anisotropic permeability.

**Figure 11:**
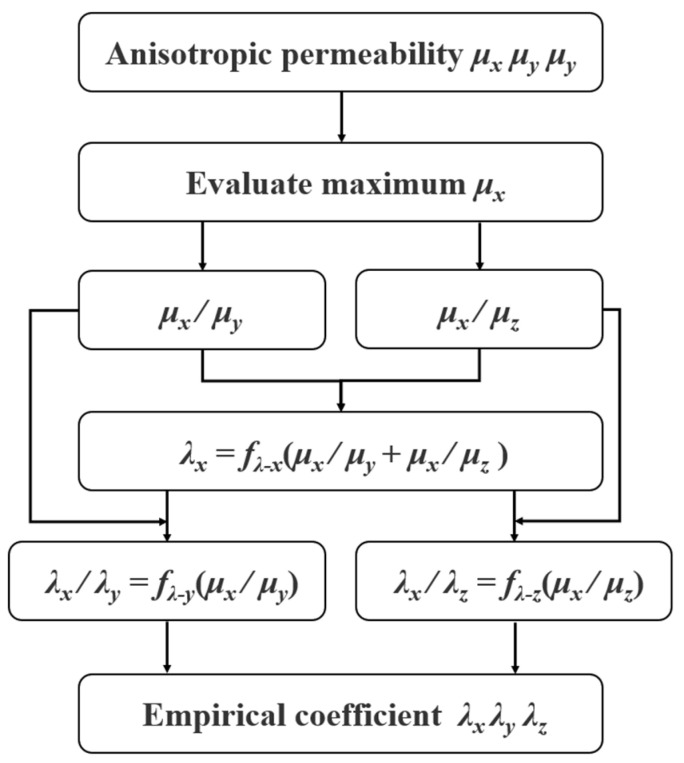
Flow chart of the calculation of empirical coefficients *λ_x_*, *λ_y_*, and *λ_z_*.

As shown in Table 2, the minimum empirical coefficient *λ_x_* and the difference in the empirical coefficients *λ_x_*, *λ_y_* and *λ_z_* depend on the disparity in anisotropic magnetic permeability across the three axes for every simulation group. Through numerical analysis, empirical coefficients *λ_x_*, *λ_y_*, and *λ_z_* can be calculated by the ratio of the maximum permeability value *μ_x_* to the two lower anisotropic permeability values *μ_y_* and *μ_z_*, in which the *λ_x_* is inversely proportional to the sum of *μ_x_*/*μ_y_* and *μ_x_*/*μ_z_*, *λ_x_*/*λ_y_* is inversely proportional to *μ_x_*/*μ_y_*, and the *λ_x_*/*λ_z_* is inversely proportional to *μ_x_*/*μ_z_*. Therefore, the flow chart for the calculation of the empirical coefficients *λ* can be shown in Figure 11.

**Figure 12:**
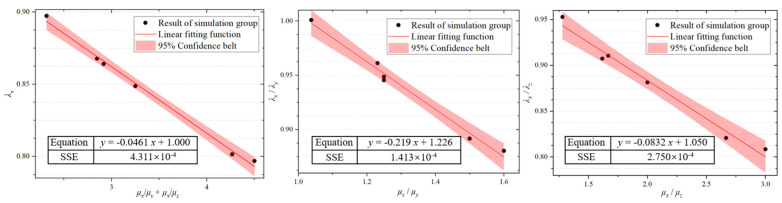
Linear fitting results of *λ_x_*, *λ_y_*, and *λ_z_*.

Mathematical fitting was processed on the six sets of data which were composed of the *λ* values obtained from simulations of argon arc welding and laser welding, along with the four sets of data presented in Table 2. The linear fitting results are shown in Figure 12; *λ_x_* and the sum of *μ_x_*/*μ_y_* and *μ_x_*/*μ_z_*, *λ_x_*/*λ_y_* and *μ_x_*/*μ_y_*, and *λ_x_*/*λ_z_* and *μ_x_*/*μ_z_* are all linearly inversely related. The six sets of simulation data are all located within the 95% confidence interval of the fitted model. The sum of squared residuals (SSE) for each fitting curve is less than 5 × 10^−4^, which demonstrates the modified model is accurate and effective. The corrected theoretical calculation formula of SF, which is introduced to the anisotropic permeability, is shown as Equation (17).

(17) $$\left[\begin{array}{c} SF_{x}\\ SF_{y}\\ SF_{z} \end{array}\right]≅1+\left[\begin{array}{c} -0.0461\left(\mu_{x}/\mu_{y}+\mu_{x}/\mu_{z}\right)+1\\ \frac{\lambda_{x}}{-0.219\mu_{x}/\mu_{y}+1.226}\\ \frac{\lambda_{x}}{-0.0832\mu_{x}/\mu_{z}+1.050} \end{array}\right]\cdot [\begin{array}{ccc} \mu_{x} & \mu_{y} & \mu_{z} \end{array}]\cdot \frac{d}{R_{1}}$$

For verifying the dependability of the modified model, an additional set of shielding factors with anisotropic permeability is calculated by the theoretical calculation formula using the modified model. Meanwhile, finite element simulation was conducted with the same anisotropic permeability, which would be compared with the theoretical calculation values to validate the credibility of the model. The anisotropic permeability is set as *μ_x_* = 40,000, *μ_y_* = 30,000, and *μ_z_* = 20,000, and the result is shown in Table 3. The finite element simulation results are used as actual observed values, the mean square error (MSE) of the original model is 49.97, while the modified model is 0.13. The calculation error of the original model is significant while processing the case of anisotropy. The modified model qualified the function to calculate the empirical coefficient, which can provide high precision calculation results for the case of anisotropy.

In addition to the aforementioned studies, this paper discusses some scenarios with potential research value. Temperature has a significant impact on magnetic properties. Sun et al. developed a system for measuring the magnetic properties of permalloy ring samples at different temperatures [30]. They measured the initial permeability of the material within a temperature range of −60 °C to 140 °C and found that it increased exponentially with rising temperature, decreasing by 69.64% at −60 °C and increasing by 38.23% at 140 °C. Therefore, the anisotropic permeability of permalloy sheets is also likely positively influenced by temperature. However, the impact on the model is minimal because temperature primarily alters the permeability. The empirical coefficient-based model can adjust *λ* for different permeability values, ensuring the accuracy of SF calculations.

In this study, the magnetic properties of permalloy sheets were measured under conditions of room temperature, no tensile stress, and intact sheet integrity. However, during the use of magnetic shielding devices, the different magnitudes and forms of stress on every surface of the shield leads to various deformations, which will cause a decrease in the permeability of permalloy sheets and a reduction in the shielding performance of the device. Prolonged use can also cause material aging and microstructural defects, all of which can alter the permeability and consequently affect the calculation of the shielding factor (SF) for the magnetic shielding region (MSR). Therefore, further investigation is necessary for permeability measurements under complex conditions such as stress–strain, operating temperature, material aging, and microstructural defects. Regarding the computational model, this study focused on a 420 × 420 × 420 mm rectangular shielding device. For shielding devices of other dimensions, there may be potential effects on the computational model and the empirical coefficient *λ*. Thus, this model warrants further research and optimization.

## 5. Conclusions

Based on the measurement results of the magnetic properties of permalloy sheets and the analysis of the shielding performance of SMRs with isotropic and anisotropic magnetic permeability, a mathematical model was established to modify the empirical coefficient λ for the theoretical calculation of SF. High-precision 2D-SST equipment was used to measure the magnetic property at a low-frequency band and various magnetization states on the directions which are parallel and perpendicular to the magnetic annealing direction, and the magnetization degree under specific excitations, basic magnetization curves, and initial magnetic permeability at more angles. The anisotropic magnetic properties of argon arc welding and laser welding Permalloy sheets were fully measured and recorded. In the shielding of the static and quasi-static magnetic field, the initial magnetic permeability, as the common reference index for shielding performance for the argon arc welding of permalloy alloys, demonstrates superior performance compared to laser welding. The anisotropic permeability of argon arc welding is 24,600 and 15,200, while the laser welding is 20,800 and 16,100. The magnetic field distribution and SFs along each axis of SMRs using isotropic and anisotropic magnetic permeability are simulated, compared, and analyzed based on measured data. The magnetic field fluctuation rate for anisotropic permeability is more than 10 times higher than that for isotropic permeability in the xoy and zox planes, and the theoretical calculation results of SFs with anisotropic permeability vary from 17.8% to 45.7%, while the SFs of isotropic permeability are uniform. Finally, according to the finite element simulation results, a modified model constructed by *λ* and *μ* was established to optimize the theoretical calculation formula of SFs, in which a linear inverse relationship among the ratio of *λ* in different directions, and the ratio of *μ* in a corresponding direction, is found. A validation group was established to conduct a comparative analysis between the original model, the modified model, and finite element simulations. The mean squared error (MSE) for the original model was 49.97, while the MSE for the modified model was 0.13. This demonstrates a substantial improvement in the accuracy of anisotropic permeability calculations with the modified model.

## Figures and Tables

**Table 1:** The main technical parameters of the tested transformer.

	Isotropy			Anisotropy	
	*μ* = 25,000	*μ* = 20,000	*μ* = 15,000	Argon Arc Welding	Laser Welding
*δ_xoy_*	10.80%	10.79%	10.77%	11.22%	10.81%
*δ_yoz_*	3.91%	3.91%	3.90%	3.57%	3.59%
*δ_zox_*	4.65%	4.64%	4.64%	4.66%	4.45%
SF_x_	110.05	88.23	66.41	102.75	89.70
SF_y_	110.04	88.22	66.41	70.36	73.76
SF_z_	109.05	87.43	65.81	86.99	86.39

**Table 2:** Simulation result of empirical coefficient λ under various anisotropic permeability.

*μ_x_*	*μ_y_*	*μ_z_*	*λ_x_*	*λ_y_*	*λ_z_*
10,000	8000	5000	0.849	0.898	0.963
30,000	20,000	10,000	0.797	0.894	0.986
50,000	40,000	30,000	0.864	0.911	0.950
80,000	50,000	30,000	0.801	0.910	0.976

**Table 3:** Simulation result of empirical coefficient λ under various anisotropic permeability.

	SF*_x_*	SF*_y_*	SF*_z_*	MSE
Original model	172.43	129.57	86.71	49.97
Modified model	162.21	130.45	92.22	0.13
Finite element simulation	161.74	130.58	92.60	

## Data Availability

The raw data supporting the conclusions of this article will be made available by the authors on request.
